# Activity of Galidesivir in a Hamster Model of SARS-CoV-2

**DOI:** 10.3390/v14010008

**Published:** 2021-12-21

**Authors:** Ray Taylor, Richard Bowen, James F. Demarest, Michael DeSpirito, Airn Hartwig, Helle Bielefeldt-Ohmann, Dennis M. Walling, Amanda Mathis, Yarlagadda S. Babu

**Affiliations:** 1 BioCryst Pharmaceuticals, Inc., 4505 Emperor Blvd. Suite 200, Durham, NC 27703, USA; JDemarest@biocryst.com (J.F.D.); MDeSpirito@biocryst.com (M.D.); DWalling@biocryst.com (D.M.W.); matamanda@gmail.com (A.M.); Babu@BIOCRYST.com (Y.S.B.); 2 Department of Biomedical Sciences, Colorado State University, Fort Collins, CO 80523, USA; rbowen@rams.colostate.edu (R.B.); airn.tolnay@colostate.edu (A.H.); 3 School of Chemistry & Molecular Biosciences, St Lucia Campus, University of Queensland, Brisbane, QLD 4072, Australia; h.bielefeldtohmann1@uq.edu.au

**Keywords:** SARS-CoV-2, nucleoside analog, coronavirus, RNA viruses, antiviral

## Abstract

Coronavirus disease 2019 (COVID-19) has claimed the lives of millions of people worldwide since it first emerged. The impact of the COVID-19 pandemic on public health and the global economy has highlighted the medical need for the development of broadly acting interventions against emerging viral threats. Galidesivir is a broad-spectrum antiviral compound with demonstrated in vitro and in vivo efficacy against several RNA viruses of public health concern, including those causing yellow fever, Ebola, Marburg, and Rift Valley fever. In vitro studies have shown that the antiviral activity of galidesivir also extends to coronaviruses. Herein, we describe the efficacy of galidesivir in the Syrian golden hamster model of severe acute respiratory syndrome coronavirus 2 (SARS-CoV-2) infection. Treatment with galidesivir reduced lung pathology in infected animals compared with untreated controls when treatment was initiated 24 h prior to infection. These results add to the evidence of the applicability of galidesivir as a potential medical intervention for a range of acute viral illnesses, including coronaviruses.

## 1. Introduction

Coronavirus disease 2019 (COVID-19) is a rapidly evolving global health emergency that has claimed the lives of more than 5 million people worldwide since it first emerged in December 2019 [1]. The disease is caused by severe acute respiratory syndrome coronavirus 2 (SARS-CoV-2) and can manifest as mild, severe, or critical disease; critical disease is associated with acute respiratory distress syndrome with respiratory failure and multi-organ failure [2]. The global pandemic caused by SARS-CoV-2 has emphasized the need to develop broadly acting antivirals effective against emerging viral diseases. The Food and Drug Administration (FDA) granted remdesivir emergency use authorization (EUA) in May 2020. In October 2020, FDA approved it for use in hospitalized patients with COVID-19 based on evidence of faster recovery times over 10 days of treatment with remdesivir compared with placebo or standard of care in phase III trials [3]. EUA was issued in November of the same year for two monoclonal antibody treatments for use in patients with mild to moderate COVID-19 at risk of progressing to severe disease and/or hospitalization [4,5]. Two oral antivirals, molnupiravir and PF-07321332/ritonavir, have shown promising results in Phase II/III trials with 50% and 89% reductions, respectively, in COVID-19-related hospitalization or death among actively treated patients compared to placebo; these are being considered for EUA [6,7].

Galidesivir is a nucleoside analog that targets the RNA-dependent RNA polymerase (RdRp) of RNA viruses [8]. Its pharmacokinetic profile has been described in several models, demonstrating initial rapid uptake of the parent compound by the cells following administration; conversion of the drug to its active triphosphate form; intracellular catabolism of the active form back to the parent compound; and slower excretion of the parent compound into the plasma [9]. Its broad-spectrum antiviral activity has been demonstrated in vitro against more than 20 RNA viruses across several viral families, including coronaviruses, filoviruses, togaviruses, phenuiviruses, pneumoviruses, arenaviruses, paramyxoviruses, orthomyxoviruses, picornaviruses, and flaviviruses [8,9]. Additionally, the clinical benefits of galidesivir have been demonstrated in vivo in animal studies of Marburg (MARV), Ebola (EBOV), Zika (ZIKV), yellow fever, and Rift Valley fever (RVFV) viruses [8,9,10,11,12,13]. Galidesivir has demonstrated moderate antiviral activity against severe acute respiratory syndrome coronavirus 1 (SARS-CoV) and Middle East respiratory syndrome coronavirus (MERS-CoV) in Vero cells; however, its activity against SARS-CoV-2 in cell culture has remained largely unexplored [8,14]. Computational modeling studies have demonstrated the potential for galidesivir to bind effectively to the RdRp of SARS-CoV-2 [15,16]. Vero cells are known to inefficiently convert galidesivir to its active form [8]. We, therefore, sought to determine both the in vitro and in vivo potency of galidesivir against SARS-CoV-2.

The Syrian golden hamster (*Mesocricetus auratus*) model is a suitable small animal model for the exploratory evaluation of effective treatments for SARS-CoV-2 infection. The model is characterized by the rapid onset of viral replication following intranasal infection, reaching peak viral titers 1–3 days post infection. Histopathological features observed in lung tissues of infected animals are similar to COVID-19-associated pneumonia in humans. SARS-CoV-2 infection typically manifests with mild clinical signs in hamsters; animals mount a neutralizing antibody response, and recovery is usually seen within two weeks of viral challenge [17,18,19,20].

## 2. Materials and Methods

### 2.1. In Vitro Evaluation of Antiviral Activity

#### 2.1.1. Cell Lines

Antiviral activity was evaluated in Caco-2 (human colon carcinoma; American Type Culture Collection (ATCC) HTB-37), Vero-76 (African green monkey (*Chlorocebus aethiops*) kidney; ATCC CRL-1587), and Calu-3 (human lung adenocarcinoma) cells.

#### 2.1.2. Test Article

Galidesivir (BCX4430) was Provided by BioCryst Pharmaceuticals, Birmingham, AL, USA and Manufactured by Millipore Sigma, Madison, WI, USA.

#### 2.1.3. Viral Yield Reduction Assay and Cytopathic Effect Assay

Antiviral activity using SARS-CoV-2 isolate USA-WA1/2020 (World Reference Center for Emerging Viruses and Arboviruses) was assessed by viral yield reduction (VYR) assay [21] on Day 3 post infection in Caco-2 and Vero-76 cells and cytopathic effect (CPE) assay [22] on Day 5 post infection in Vero-76 cells. Antiviral activity was assessed with eight serial dilutions of galidesivir, ranging from 0.032 to 100 µg/mL. M128533, a protease inhibitor with known antiviral activity against SARS-CoV and SARS-CoV-2, was included as a positive control. Twenty-four hours after incubation with galidesivir, Caco-2 and Vero-76 cells were infected with SARS-CoV-2 at a multiplicity of infection (MOI) of 0.02 and 0.002, respectively. Cytotoxicity was assessed using the neutral red cytopathic assay. The 50% effective concentration (EC_50_) and 90% effective concentration (EC_90_) were calculated by linear regression.

#### 2.1.4. In Vitro Imaging Assay

An in vitro imaging assay using SARS-CoV-2 isolate USA-WA1/2020 (BEI Resources) and Calu-3 cells was performed as previously described [23]. Briefly, Calu-3 cells were plated in 384-well plates. The following day antiviral activity was assessed in duplicates of eight serial dilutions of galidesivir, ranging from 0.023 to 50.0 µM. DMSO was included as a negative control, and remdesivir was included as a positive control. Two hours after incubation with galidesivir, cells were infected with SARS-CoV-2 under biosafety level 3 (BSL3) containment conditions at an MOI of 0.5. Forty-eight hours after viral infection, cells were fixed and incubated overnight at 4 °C with a primary antibody specific for dsRNA (anti-dsRNA J2). Following 1 h incubation with a secondary antibody at room temperature, the cells were processed for automated microscopy. Toxicity was measured by quantifying the number of cells per well. The percentage of infected cells was calculated using the formula: (dsRNA^+^ cells/cell number)/well.

### 2.2. In Vivo Evaluation of Galidesivr in the Hamster Model of SARS-CoV-2

#### 2.2.1. Animals

Male and female Syrian golden hamsters (Envigo Corp., Indianapolis, IN, USA) were housed in ventilated cages under BSL3 containment at Colorado State University. Hamsters were randomly removed from their shipping containers and placed into cages on arrival. Animals were approximately eight weeks old at the start of the experiments, with an average weight of ~145 g. All animals were implanted with a thermal-sensitive microchip during the 7-day acclimatization period prior to the virus challenge for identification and daily monitoring of weight.

#### 2.2.2. Test Article and Vehicle Control

Galidesivir (BCX4430) dihydrochloride (BioCryst Pharmaceuticals, Inc., Durham, NC, USA) was supplied as a powder and reconstituted in lactated Ringer’s Injection United States Pharmacopeia (USP). The 77.5% BCX4430 dihydrochloride solution was diluted to achieve treatment doses of 100 mg/kg in volumes of 0.1 and 0.2 mL for administration by intraperitoneal injection. Lactated Ringer’s Injection USP was used as the vehicle control.

#### 2.2.3. Virus

The WA1/2020 isolate of SARS-CoV-2 was passaged twice in Vero E6 cells after its receipt from BEI Resources (BEI Resources, item FSCUST-85), and frozen stocks were prepared and titrated.

#### 2.2.4. Experimental Infection and Evaluation of Syrian Golden Hamsters

Hamsters were divided into four groups of eight animals each, with equal numbers of male and female animals in each group. A viral challenge with SARS-CoV-2 was conducted under ketamine-xylazine anesthesia by intranasal instillation of 100 µL of the virus suspension for a target dose of 1 × 10^4^ plaque-forming units (PFU).

Animals were treated by intraperitoneal injection with 100 mg/kg galidesivir two times per day (BID) at 12 h intervals for 6, 7, or 8 days, depending on when the first injection was administered relative to infection. The first dose was administered 24 h pre infection (Group 1), 1.5 h post infection (Group 2), or 24 h post infection (Group 3). The control group (Group 4) was injected BID with the vehicle.

Clinical evaluation of the animals was conducted daily, including measurement of body weight and clinical score.

Oropharyngeal (OP) swabs were collected on Days 1, 2, and 3 post infection by rotating a polyester swab in the oral cavity and pharynx of animals for approximately 5 s. Swabs were then placed in 1 mL of BA1 (Tris-buffered minimum essential medium containing 1% bovine serum albumin with 10% fetal bovine serum) and kept at −80 °C until analysis.

On Day 3 and Day 7 post infection, four animals per group were euthanized by administering an overdose of ketamine-xylazine followed by cervical dislocation. From hamsters euthanized on Day 3, turbinate, cranial, and caudal lobes of lung tissue were collected and homogenized using a Qiagen Tissuelyser at 25 cycles per second for 5 min. Homogenates were kept frozen until viral titer analysis. Turbinate, trachea, lung, and heart tissues were collected from animals euthanized on Day 3 and Day 7, fixed in neutral-buffered formalin, and processed to obtain standard hematoxylin- and eosin-stained sections for histopathologic evaluation by a blinded veterinary pathologist using a previously outlined scoring system [24].

#### 2.2.5. Virus Plaque Assay

Virus titrations were conducted using a double-overlay plaque assay [25] on Vero E6 cells cultured in six-well plates. Cells were propagated in Dulbecco’s minimal essential medium supplemented with 5% fetal bovine serum, and all incubations were at 37 °C. Samples of oral swab fluid or 10% suspensions of tissue homogenates were serially diluted in BA1, and 100 µL of each dilution was inoculated onto each well. Plaques were read 2 and 3 days later. The limit of detection of the assay was 10 PFU/100 mg or 10 PFU per swab. For calculating means and summary statistics, undetectable readings were converted to 5 PFU/100 mg or 5 PFU per swab.

#### 2.2.6. Statistical Analysis

Analysis of covariance was used to assess differences in adjusted least-squares means from baseline (Day 1) at Days 2 through 7 between active treatment groups and the control group. Multiple comparisons of mean differences in histopathology score and viral burden between active treatment groups and the control group were assessed by one-way analysis of variance with Dunnett’s test. Differences in frequencies of undetectable viral burden and no detectable tissue damage between active treatment groups and the control group were assessed by chi-square statistics. Pearson’s correlation was used to assess the relationship between viral burden and total histopathology score. The significance threshold for all tests was set at the 0.05 level. No formal hypothesis testing was conducted.

## 3. Results

### 3.1. Evaluation of the In Vitro Antiviral Activity of Galidesivir against SARS-CoV-2

The antiviral activity of galidesivir in cell culture was assessed at three independent laboratories using separate assay systems for evaluation. Galidesivir was found to be active in SARS-CoV-2 infected Caco-2 and Vero-76 cells as assessed by VYR assay, with low EC_90_ values for both cell lines and favorable selectivity index (SI) values (Table 1). Galidesivir was also evaluated in Vero-76 cells using a CPE assay; the reported EC_90_ value was notably higher with the CPE assay than with the VYR assay (Table 1). This discrepancy is potentially a consequence of different measures of antiviral activity among the two assay systems and a 2-days-later readout of results for the CPE assay compared with the VYR assay. Galidesivir was also demonstrated to be active in SARS-CoV-2 infected Calu-3 cells, evaluated using an imaging assay (Table 1).

### 3.2. Evaluation of the In Vivo Activity of Galidesivr in the Hamster Model of SARS-CoV-2

#### 3.2.1. Study Design

**Figure 1:**
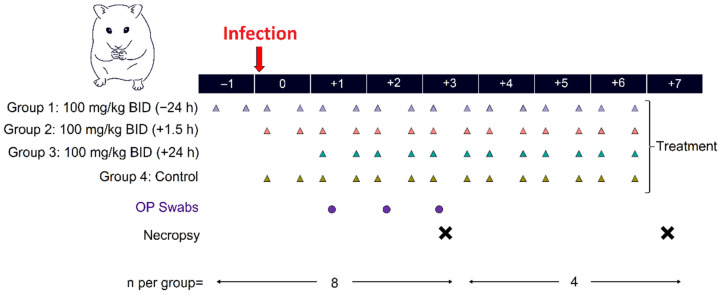
Study design. SARS-CoV-2 infected animals were treated twice a day (BID) by intraperitoneal injection with galidesivir or the vehicle control. Oropharyngeal (OP) swabs were collected on Days 1, 2, and 3 post infection. Lung, turbinate, trachea, and heart tissues were collected on Days 3 and 7 post infection. There were eight animals per group; four animals per group were euthanized on Day 3, and the remaining four were euthanized on Day 7. Triangles represent schedule for BID treatments for Group 1 (lilac), Group 2 (orange), Group 3 (teal), and Control (brown). Purple circles represent OP swabs. Black X represents necropsy.

The aim of this study was to evaluate the efficacy of galidesivir at a dosage of 100 mg/kg BID in reducing viral replication and mitigating pulmonary pathology in Syrian golden hamsters with treatment initiation at three timepoints relative to SARS-CoV-2 infection (Figure 1). Animals in Group 1 received their first dose 24 h prior to infection, animals in Group 2 received their first dose 1.5 h post infection, and animals in Group 3 received their first dose 24 h post infection. Treated animals were compared with a control group that received BID injections of lactated Ringer’s USP.

#### 3.2.2. Clinical Evaluation

**Figure 2:**
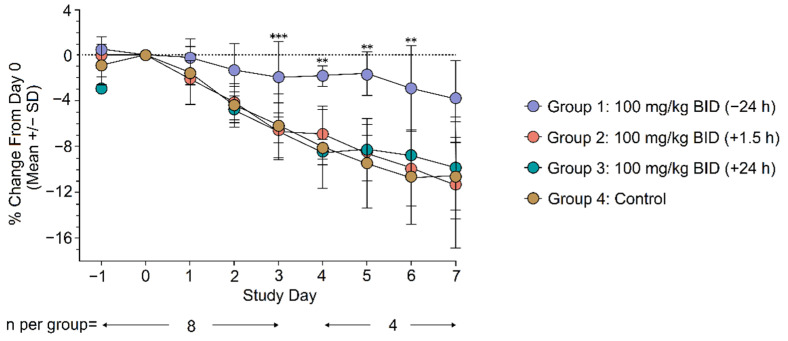
Mean weight change from Day 0 through Day 7. Statistical analysis was conducted using an ANCOVA model with terms for treatment and baseline value as covariates. ** *p* < 0.01, *** *p* < 0.001 for difference in adjusted least-square means compared with the control. SD, standard deviation; BID, twice daily dosing.

The body weight of animals was monitored daily from 2 days prior to viral challenge until euthanasia. Weight change from Day 0 (day of infection) was evaluated daily across treatment groups as a surrogate for antiviral activity. Animals treated with galidesivir 100 mg/kg BID initiated 24 h prior to SARS-CoV-2 infection (Group 1) exhibited significantly less weight loss compared with the control group at Days 3 to 6 post infection (Day 3, *p* = 0.0004; Day 4, *p* = 0.002; Day 5, *p* = 0.001; Day 6, *p* = 0.009; Figure 2). No differences in weight change were observed between animals in Group 2 and Group 3 compared with the control group (Figure 2). Daily clinical scores were similar across all groups, and a large majority of animals did not show signs of overt clinical disease (data not shown).

#### 3.2.3. Viral Burden

Replication-competent SARS-CoV-2 was quantified in OP swabs collected on Days 1, 2, and 3 post infection and turbinate and lung tissues collected on Day 3 post infection.

**Figure 3:**
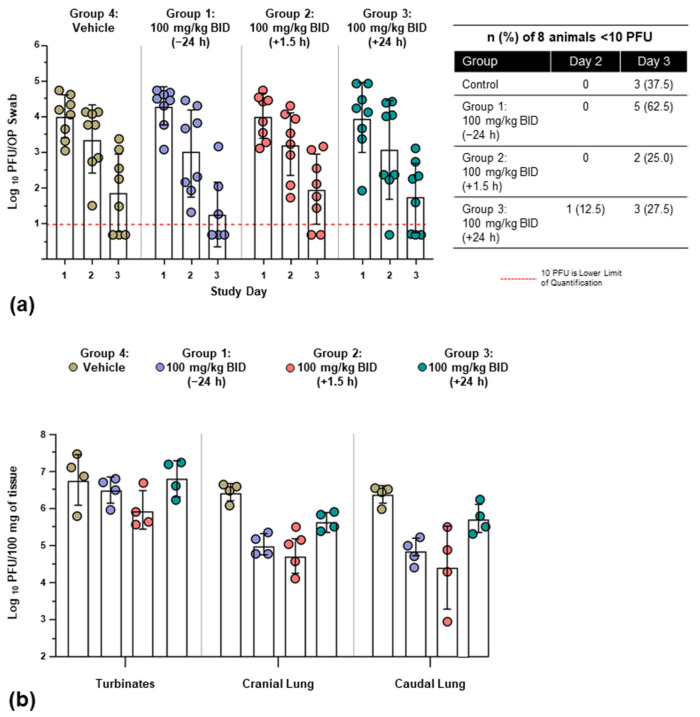
Mean viral burden (log_10_) in OP swabs, turbinate, and lung tissues. (**a**) Replicative virus was detected by OP swabs from Day 1 through Day 3 across treatment groups. (**b**) Replicative virus in turbinate and lung tissues on Day 3 post infection. All groups receiving active treatment had lower viral burden in lung tissues relative to controls. Analysis of multiple comparisons was conducted with a One-way ANOVA with Dunnett’s test. PFU, plaque-forming unit; BID, twice daily dosing.

All animals shed virus from Day 1 through Day 3 post infection; the peak viral burden in OP swabs was observed at Day 1 and decreased over the course of the sampling period across all groups (Figure 3a). Animals in Group 1 experienced a numerically greater reduction in viral burden from Day 1 to Day 3 compared with the three other groups; however, differences were not statistically significant. By Day 3, five animals (62.5%) in Group 1 had no detectable virus in OP swabs, compared with two animals (25.0%) in Group 2 and three animals (37.5%) in both Group 3 and the control group; however, these numerical differences did not reach statistical significance.

A reduced viral burden in cranial and caudal lung samples was seen across all galidesivir-treated animals relative to control animals (Figure 3b), with statistically significant mean differences observed in Group 1 and Group 2 (Table 2). Animals in Group 1 and Group 2 also exhibited lower viral burden in turbinate tissue compared with controls, although the differences were not statistically significant (Figure 3b; Table 2).

**Figure 4:**
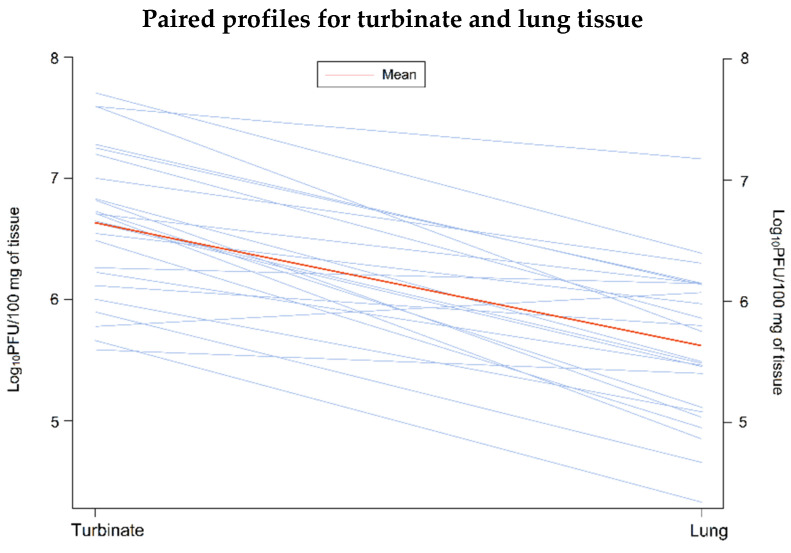
Paired profiles of viral burden in turbinate and lung tissue (n = 24). The analysis was conducted using a paired T-test, which compared the mean viral burden in turbinate and average lung tissue (average of cranial and caudal lung samples) from the same hamster. PFU, Plaque forming units.

The relative viral burden in turbinate and lung tissues (average of cranial and caudal lung samples) was compared for individual animals. This analysis showed that the mean viral burden was significantly lower in lung tissue compared with turbinate tissue among animals receiving active treatment (n = 24, mean difference = 1.009; *p* < 0.0001; Figure 4). A similar difference in viral load between the lower and upper respiratory tract was not observed in the four control animals.

#### 3.2.4. Histopathology

Histopathology scores were used to quantify the degree of tissue injury in individual animals, providing a measure for quantifying the protective effect of galidesivir. Scoring was performed by a blinded pathologist on a scale of 0–5, with higher scores indicative of more severe tissue damage [24]. Histopathology of the lungs, trachea, and heart was assessed on Day 3 and Day 7, with four animals per treatment group evaluated at each time point.

**Figure 5:**
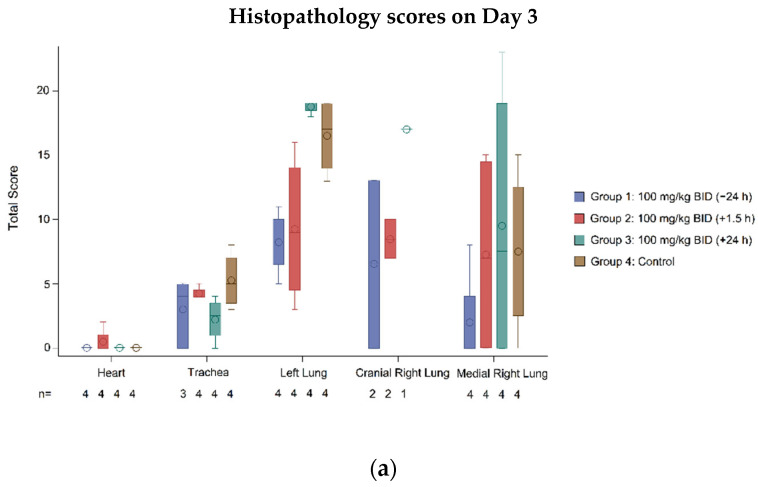

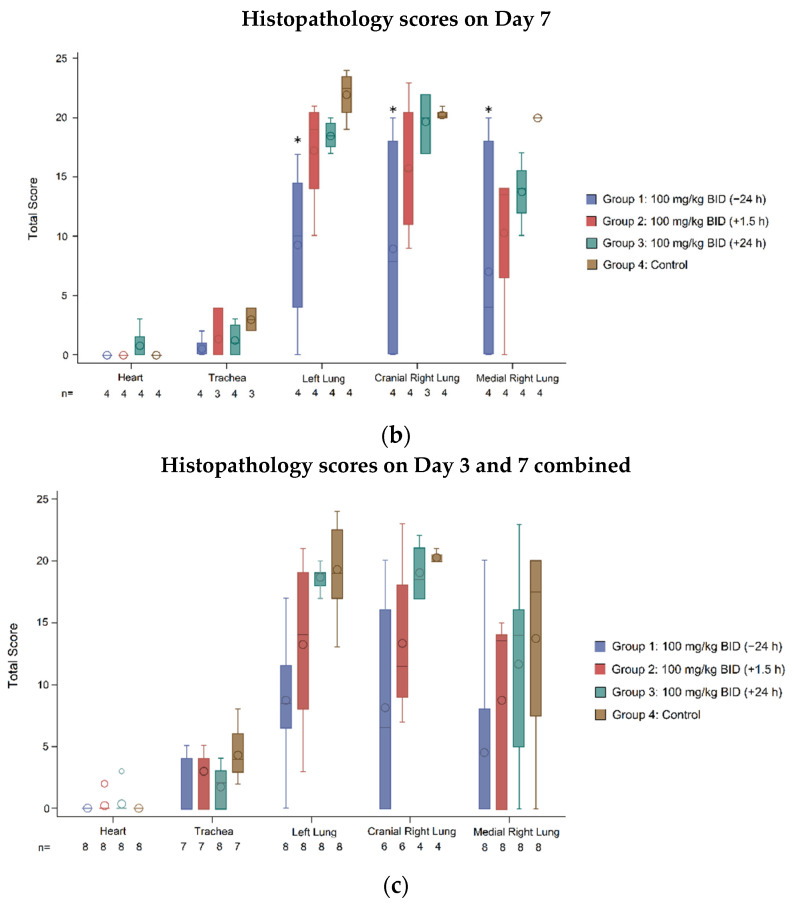
Histopathology. Boxplots represent median, Q1, and Q3. The mean is indicated by a circle within the boxplot, and whiskers extend to minimum and maximum values. (**a**) Histopathology scores from lung, trachea, and heart tissues at Day 3 post infection. (**b**) Histopathology scores from lung, trachea, and heart tissues at Day 7 post infection. (**c**) Histopathology scores from lung, trachea, and heart tissues at Day 3 and Day 7 post infection combined. Statistical significance was tested using Dunnetts’ test for mean differences. * *p* < 0.05 compared to control. BID, twice daily dosing.

Lung tissue histology indicated a protective effect of galidesivir when initiated 24 h prior to infection (Group 1), with lower histopathology scores in this group compared with the control (Figure 5a,b). Overall, when combining results for Day 3 and Day 7, a significant difference in means was detected between Group 1 and the control for the left lung (difference vs. control: −10.5, 95% CI (−17.67 to −3.34); *p* < 0.05; Figure 5c) and cranial right lung (difference vs. control: −12.1, 95% CI (−23.21 to −0.96); *p* < 0.05; Figure 5c) but not for the medial right lung (difference vs. control: −9.3, 95% CI (−18.90 to 0.40); Figure 5c). Animals in Group 2 also exhibited lower histopathology scores than control animals but averaged higher scores compared with animals in Group 1. From Day 3 to Day 7, there was a trend for worsening histopathology scores among animals in the control group and Group 2. This trend was not observed over the same period for animals in Group 1; however, greater variability in outcomes was evident in this group of animals on Day 7 compared with Day 3. The within-group variability in histopathology scores for animals in both Group 1 and Group 2 was relatively high (Figure 5); as such, representative histopathology images from these groups could not be obtained. Galidesivir treatment initiated 24 h post infection (Group 3) did not provide measurable protection from lung histopathology compared with the control group.

Infection-mediated tissue damage was also assessed in the trachea and heart. In tracheal tissue, galidesivir provided the greatest protection for animals when treatment was initiated earlier, demonstrated by lower histopathology scores in Group 1 relative to the other groups at Day 7 (Figure 5b). Additionally, a significantly greater number of animals in Group 1 presented with no observable histopathology compared with the control (Group 1 = 4 (50.0%), control = 0; *p* = 0.02; Table 3). Across all groups, there was a trend for decreased pathology in tracheal tissue from Day 3 to Day 7 (Figure 5a,b).

Apart from one outlier in Group 2 and Group 3, respectively, SARS-CoV-2 infection was not associated with observable heart pathology (Figure 5).

#### 3.2.5. Association of Viral Burden and Histopathology Scores

**Figure 6:**
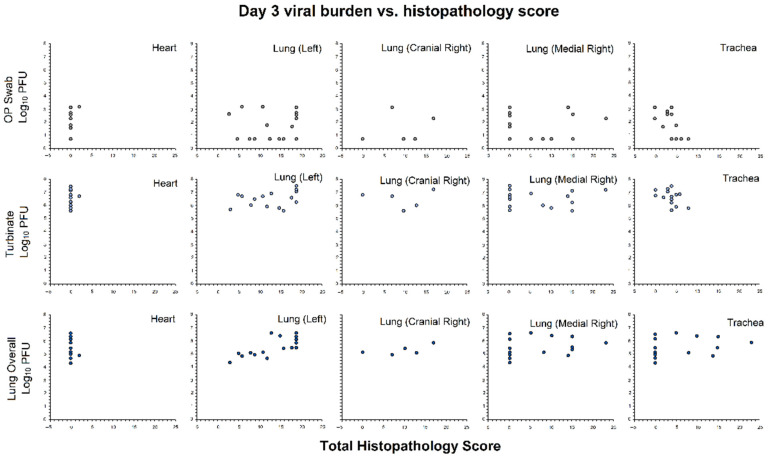
Correlation analysis. Pearson’s correlation was used to measure the association of viral burden and histopathology total scores at each location for hamsters with both histopathology data and matching viral burden data at Day 3 (n = 16). Viral burden data were collected from OP swabs, lungs (combined cranial and caudal), and turbinates. Grey, OP swabs; Light blue, turbinates; Dark blue, lungs.

For hamsters with histopathology and viral burden data at Day 3 (n = 16), a correlation analysis was conducted to determine if there was an association between levels of viral burden and tissue pathology (Figure 6). A significant negative association was observed between the viral burden in OP swabs and total histopathology scores from tracheal tissue (higher PFU was associated with lower histopathology scores, *r* = −0.55; *p* = 0.03), and a significant positive association was observed between the average viral burden in lungs (combined cranial and caudal) and total histopathology scores from left lung tissues (higher PFU was associated with higher histopathology scores, *r* = 0.73; *p* = 0.001). No significant association was identified between the viral burden in turbinates and tracheal, heart, or lung histopathology scores.

## 4. Discussion

The in vitro antiviral activity of galidesivir against SARS-CoV-2 was demonstrated by three independent laboratories. The reported SI values were favorable, ranging from >3.5 to >27 depending on the assay system used. The potency of galidesivir observed across these analyses was comparable to that previously reported for galidesivir against SARS-CoV and more potent than that previously reported for galidesivir against MERS-CoV [8]. The activity of galidesivir against SARS-CoV-2, SARS-CoV, and MERS-CoV tested in Vero cells may underestimate its antiviral potency against coronaviruses due to the inefficient conversion of galidesivir to its active triphosphate form within these cell lines [13]. The efficiency at which Caco-2 and Calu-3 cells convert galidesivir to its active form is not known. Although antiviral activity was demonstrated for galidesivir in Calu-3 cells, it must be noted that the protocol used had a substantially shorter pre-incubation period than what is recommended for galidesivir in cell culture. The recommended pre-incubation period for galidesivir is 24 h; however, galidesivir was pre-incubated with Calu-3 cells for only 2 h prior to infection. As such, the activity of galidesivir tested by this assay system may be underestimated. The effect of suboptimal pre-incubation was seen in a previously published in vitro study, which reported that no activity was observed for galidesivir against SARS-CoV-2 in Vero-6 cells pre-incubated with galidesivir for 1 h [14].

In the hamster model of SARS-CoV-2 infection, early treatment with galidesivir (100 mg/kg BID; first injection administered 24 h before viral challenge) was associated with clinical and virologic benefits. Animals in this treatment group experienced reduced weight loss, reduced viral burden, and reduced tissue pathology compared with control animals and compared with animals that received galidesivir treatment initiated at later timepoints relative to infection. The antiviral efficacy and tissue-protective effects were most clearly observed in lung tissues; this outcome is relevant to note because an important desired effect of antiviral treatment in human COVID-19 cases would be to prevent or reduce the progression of virus-induced lung disease. Galidesivir (100 mg/kg BID) initiated 1.5 h post infection was associated with some clinical and virologic benefits compared with the control, although to a lesser extent than animals that received galidesivir treatment prior to infection. Galidesivir (100 mg/kg BID) initiated 24 h post infection did not provide any benefit to mitigate infection in this study compared with the control. The lack of benefit in the 24 h post infection treatment group may be explained by the kinetics of the model, in which peak viral replication occurred at Day 1 post infection, coinciding with the initiation of treatment in this group of animals. Similarly, the rapid onset of viral replication and peak viral load could explain the lower efficacy of treatment in the 1.5 h post-infection treatment group relative to the 24 h pre-infection treatment group. These results suggest that galidesivir treatment of SARS-CoV-2 infection should be initiated prior to or at the time of infection in this animal model to increase the likelihood of providing clinical benefit. Similarly, pre-infection treatment of Syrian hamsters with a small molecule inhibitor targeting the SARS-CoV-2 main protease was recently shown to inhibit viral replication in lung tissue [26]. In addition, in a rhesus macaque primate model, early initiation of treatment at 12 h after SARS-CoV-2 infection with remdesivir, an adenosine analog antiviral similar to galidesivir, similarly resulted in greater antiviral effect in the lungs as compared to the upper respiratory tract and less virus-induced lung tissue pathology [27]. A greater clinical benefit with early treatment initiation has also been observed with other medical countermeasures for COVID-19 as well as other viral infections treated with antivirals, such as influenza [5,28,29].

Previous animal studies have demonstrated the efficacy of using a loading-dose strategy with galidesivir against EBOV, ZIKV, and RVFV [11,12,30]. In the peracute and uniformly lethal Syrian golden hamster model of RVFV, a galidesivir treatment regimen encompassing a loading dose of 400 mg/kg administered 30 min prior to infection followed by a maintenance dose of 100 mg/kg BID resulted in a survival rate of 70% [12]. In a non-human primate model of EBOV disease, a regimen of a 100 mg/kg loading dose followed by a 25 mg/kg BID maintenance dose resulted in 100% survival, even when treatment was delayed up to 2 days [30]. A similar regimen of a 100 mg/kg loading dose followed by a 25 mg/kg BID maintenance dose prevented or rapidly reduced viral burden in a non-lethal rhesus macaque model of ZIKV [11]. The efficacy of delayed treatment with galidesivir when using a regimen that includes a loading dose may be enhanced by the rapid attainment of steady-state exposure levels through a single administration of a relatively high dose, which is subsequently safely maintained with serial injections at a lower dose quantity. It would, therefore, be interesting to investigate the efficacy of a loading-dose strategy with galidesivir in the SARS-CoV-2 hamster model, especially to investigate the potential to obtain a greater therapeutic effect with delayed treatment initiation.

Across all groups, galidesivir appeared to be well-tolerated; however, a cohort of uninfected animals receiving galidesivir treatment was not included in this study to verify this observation. The maximum tolerated dose of galidesivir in Syrian golden hamsters was previously reported as 200 mg/kg BID for 7 days in a study evaluating galidesivir for the treatment of yellow fever [31]. Exploring an increase in the dose of galidesivir from 100 mg/kg BID to 150 mg/kg BID or 200 mg/kg BID could result in a more pronounced clinical effect against SARS-CoV-2 in this animal model. As seen with the RVFV hamster model, a dose exceeding 200 mg/kg could safely be administered as a single injection in the context of a loading-dose strategy [12].

The main limitation of this study is its small sample size, and all reported outcomes must be carefully considered. Due to the small number of samples, discrepancies in statistical analyses may have arisen because of a lack of power and high variability within groups. In addition to the small sample size, another factor that may have caused increased within-group variability is the use of both male and female hamsters.

Overall, the above results provide support for further investigation of galidesivir as a treatment for SARS-CoV-2 infection. Furthermore, these outcomes add to the evidence of the broad-spectrum antiviral activity of galidesivir against RNA viruses and the role of galidesivir as a potential tool against a wide range of emerging infectious threats.

## Figures and Tables

**Table 1:** Galidesivir activity in cell culture.

Cell Line	SARS-CoV-2 Strain	Assay	Compound Pre-Incubation Period	EC_50_ (μM)	EC_90_ (μM)	CC_50_ (μM)	SI
Caco-2	WA1/2020	VYR	24 h	n.d.	14.19	82.8	5.8 *
Vero-76	WA1/2020	VYR	24 h	n.d.	10.94	>295.7	>27 *
		CPE			50.3		5.8 *
Calu-3	WA1/2020	Imaging	2 h ^‡^	14.15	n.d.	>50	>3.5 ^†^

* SI_90_; ^†^ SI_50_; ^‡^ The recommended period for pre-incubation with galidesivir is 24 h. CC, cytopathic concentration; CPE, cytopathic effect; EC, effective concentration; n.d., not determined; SARS-CoV-2, severe acute respiratory syndrome coronavirus 2; SI, selectivity index; VYR, viral yield reduction.

**Table 2:** Mean difference (log_10_) in viral burden at Day 3 post infection in caudal lung, cranial lung, and turbinate tissues between groups receiving active galidesivir treatment and the vehicle control.

	Group	n	Mean (PFU/100 mg, log_10_)	Difference vs. Control	95% CI for Treatment Difference	*p*-Value ^1^
Caudal lung	Group 1: 100 mg/kg BID (−24 h)	4	4.86	−1.53	(−2.68, −0.38)	<0.05
	Group 2: 100 mg/kg BID (+1.5 h)	4	4.42	−1.97	(−3.12, −0.81)	<0.05
	Group 3: 100 mg/kg BID (+24 h)	4	5.74	−0.65	(−1.80, 0.51)	NS
	Group 4: control	4	6.39	NA	NA	NA
Cranial lung	Group 1: 100 mg/kg BID (−24 h)	4	5.03	−1.42	(−2.22, −0.61)	<0.05
	Group 2: 100 mg/kg BID (+1.5 h)	4	4.75	−1.70	(−2.50, −0.89)	<0.05
	Group 3: 100 mg/kg BID (+24 h)	4	5.68	−0.77	(−1.57, 0.04)	NS
	Group 4: control	4	6.45	NA	NA	NA
Turbinates	Group 1: 100 mg/kg BID (−24 h)	4	6.51	−0.31	(−1.47, 0.86)	NS
	Group 2: 100 mg/kg BID (+1.5 h)	4	5.96	−0.85	(−2.02, 0.31)	NS
	Group 3: 100 mg/kg BID (+24 h)	4	6.83	0.01	(−1.15, 1.18)	NS
	Group 4: control	4	6.81	NA	NA	NA

^1^ Dunnett’s test for mean differences. CI, confidence interval; NA, not applicable; NS, not significant; PFU, Plaque Forming Units; BID, twice daily dosing.

**Table 3:** Frequency of occurrence of total histopathology scores of zero by treatment group (combined scores for Day 3 and Day 7).

Tissue	Statistic	Group 1: 100 mg/kg BID (−24 h)	Group 2: 100 mg/kg BID (+1.5 h)	Group 3: 100 mg/kg BID (+24 h)	Group 4: Control
Heart	Animals with score of zero, n (%)	8 (100)	7 (87.5)	7 (87.5)	8 (100)
	*p*-value ^1^ vs. control	NA	0.3017	0.3017	
Trachea	Animals with score of zero, n (%)	4 (50.0)	2 (25.0)	3 (37.5)	0
	*p*-value ^1^ vs. control	0.0209	0.1306	0.0547	
Left lung	Animals with score of zero, n (%)	1 (12.5)	0	0	0
	*p*-value ^1^ vs. control	0.3017	NA	NA	
Cranial right lung	Animals with score of zero, n (%)	3 (37.5)	0	0	0
	*p*-value ^1^ vs. control	0.0547	NA	NA	
Medial right lung	Animals with score of zero, n (%)	5 (62.5)	3 (37.5)	2 (25.0)	1 (12.5)
	*p*-value ^1^ vs. control	0.0389	0.2482	0.5218	NA

^1^ *p*-value based on chi-square statistics. BID, Twice daily dosing; NA, Not applicable.

## Data Availability

Restrictions may apply to the availability of these data as the data were generated through a multi-party agreement.
